# Elevated SAA1 promotes the development of insulin resistance in ovarian granulosa cells in polycystic ovary syndrome

**DOI:** 10.1186/s12958-021-00873-3

**Published:** 2022-01-03

**Authors:** Qinling Zhu, Yue Yao, Lizhen Xu, Hasiximuke Wu, Wangsheng Wang, Yaqiong He, Yuan Wang, Yao Lu, Jia Qi, Ying Ding, Xinyu Li, Jiaan Huang, Hanting Zhao, Yanzhi Du, Kang Sun, Yun Sun

**Affiliations:** 1 Center for Reproductive Medicine, Ren ji Hospital, School of Medicine, Shanghai Jiao Tong University, Shanghai, 200135 People’s Republic of China; 2 Shanghai Key Laboratory for Assisted Reproduction and Reproductive Genetics, Shanghai, 200135 People’s Republic of China

**Keywords:** Serum amyloid A1, Polycystic ovary syndrome, Insulin resistance, Granulosa cells, Inflammation

## Abstract

**Background:**

Insulin resistance (IR) contributes to ovarian dysfunctions in polycystic ovarian syndrome (PCOS) patients. Serum amyloid A1 (SAA1) is an acute phase protein produced primarily by the liver in response to inflammation. In addition to its role in inflammation, SAA1 may participate in IR development in peripheral tissues. Yet, expressional regulation of SAA1 in the ovary and its role in the pathogenesis of ovarian IR in PCOS remain elusive.

**Methods:**

Follicular fluid, granulosa cells and peripheral venous blood were collected from PCOS and non-PCOS patients with and without IR to measure SAA1 abundance for analysis of its correlation with IR status. The effects of SAA1 on its own expression and insulin signaling pathway were investigated in cultured primary granulosa cells.

**Results:**

Ovarian granulosa cells were capable of producing SAA1, which could be induced by SAA1 per se. Moreover, the abundance of SAA1 significantly increased in granulosa cells and follicular fluid in PCOS patients with IR. SAA1 treatment significantly attenuated insulin-stimulated membrane translocation of glucose transporter 4 and glucose uptake in granulosa cells through induction of phosphatase and tensin homolog deleted on chromosome 10 (PTEN) expression with subsequent inhibition of Akt phosphorylation. These effects of SAA1 could be blocked by inhibitors for toll-like receptors 2/4 (TLR 2/4) and nuclear factor kappa light chain enhancer of activated B (NF-κB).

**Conclusions:**

Human granulosa cells are capable of feedforward production of SAA1, which significantly increased in PCOS patients with IR. Excessive SAA1 reduces insulin sensitivity in granulosa cells via induction of PTEN and subsequent inhibition of Akt phosphorylation upon activation of TLR2/4 and NF-κB pathway. These findings highlight that elevation of SAA1 in the ovary promotes the development of IR in granulosa cells of PCOS patients.

**Supplementary Information:**

The online version contains supplementary material available at 10.1186/s12958-021-00873-3.

## Background

Polycystic ovarian syndrome (PCOS) is a complex endocrine and metabolic disease, which affects up to 7-12% reproductive-aged women [1]. The key characteristics of PCOS include anovulation, hyperandrogenism, polycystic ovarian morphology and insulin resistance (IR) [2, 3]. The prevalence of IR is up to 50-70% in PCOS patients [4, 5], which is found in a number of tissues and cells including ovarian granulosa cells [6, 7]. Given the essential roles of granulosa cells in folliculogenesis, IR of granulosa cells is a contributor to reproductive disorders in PCOS patients. Thus, elucidation of the mechanism underlying IR development in granulosa cells may help better understanding the pathogenesis of PCOS.

Accumulating evidence has indicated a strong association of low-grade chronic inflammation with the development of IR in peripheral tissues [8–10]. Serum amyloid A (SAA), an acute phase protein, is produced mainly by the liver in response to inflammation and trauma. Although the exact role of SAA in the acute phase response is not fully understood, SAA is at least known to participate in inflammatory reactions through activation of toll-like receptor 2 and 4 (TLR2/4) and nuclear factor kappa-light-chain-enhancer of activated B cells (NF-κB) signaling pathway [11, 12]. There are four *SAA* genes, *SAA1-4.* While *SAA1* and *SAA2* are inducible in inflammation, *SAA4* is constitutively expressed and *SAA3* is a pseudogene in humans [13]. Of interest, expression of SAA has been detected in non-hepatic tissues and its over-expression has been implicated in chronic inflammation and IR development in peripheral tissues in obesity and type 2 diabetes [14–17]. *SAA1, SAA2 and SAA4* transcripts have also been detected in human ovarian granulosa cells with *SAA1* being the most abundant isoform [18]. However, very little is known about the expression and production regulation of SAA1 in ovarian granulosa cells. It is neither known whether its over-production is associated with the development of IR through activation of the inflammatory pathway in granulosa cells in PCOS.

It is well known that insulin stimulates glucose uptake via induction of glucose transporter (GLUT4) translocation from cytoplasm to cell membrane upon activation of Akt phosphorylation [19, 20]. This signaling pathway can be attenuated by phosphatase and tensin homolog deleted on chromosome 10 (PTEN) [21, 22] and upregulation of PTEN expression has been shown to be responsible for insulin resistance in a number of tissues [23–25]. Thus, we hypothesize that increased SAA1 production may inhibit insulin-induced Akt phosphorylation via stimulation of PTEN expression upon activation of TLR2/4 and NF-κB pathway, resulting in the attenuation of insulin-induced GLUT4 translocation, glucose uptake in granulosa cells and IR development in granulosa cells in PCOS. Herein, we examined the hypothesis by using human ovarian granulosa cells obtained from PCOS and non-PCOS patients with or without IR.

## Methods

### Recruitment of patients and collection of blood, follicular fluid and ovarian granulosa cells

Blood, follicular fluid and ovarian granulosa cells were collected from patients who underwent in vitro fertilization (IVF) or intracytoplasmic sperm injection (ICSI) at Center for Reproductive Medicine between January 2018 and December 2020. Renji Hospital of Shanghai Jiao Tong University under a protocol approved by the Ethics Committee of Renji Hospital of Shanghai Jiao Tong University with informed consent (No. 2018072606). PCOS was diagnosed according to the revised Rotterdam consensus [26]. Women with tubal factor-related infertility were enrolled into the non-PCOS group. Enrolled PCOS and non-PCOS patients were sub-divided into groups with and without IR according to HOMA-IR (homeostasis model of assessment for insulin resistance index), which was calculated following the equation of fating insulin×fasting glucose/22.5). HOMA-IR ≥ 2.68 was diagnosed as IR [27]. Four groups were thus classified as following: non-PCOS without IR (*n* = 22), non-PCOS with IR (*n* = 22), PCOS without IR (*n* = 22) and PCOS with IR (*n* = 22). Insulin and glucose levels in the blood were measured with chemiluminescence assay (Beckman Access Health Co) and glucose oxidase method (Roche, Mannheim, Germany) respectively. Subjects with any of the following were excluded from this study: (1) history of unilateral oophorectomy; (2) endometriosis; (3) abnormal karyotype; (4) recurrent spontaneous miscarriage; and (5) other medical conditions such as acute inflammation screened by body temperature, blood routine test and high-sensitive C-reactive protein (CRP) test that contraindicated assisted reproductive technology and/or pregnancy. Patients diagnosed with IR were prescribed metformin treatment before IVF or ICSI treatment.

GnRH antagonist protocol was adopted for ovarian stimulation in both PCOS and non-PCOS patients under routine protocolperformed in our reproductive center. When two or more oocytes measured greater than 18 mm, urinary human chorionic gonadotropin was administered to trigger ovulation. Oocytes retrieval was performed 34-36 h later. Retrieved eggs were fertilized by IVF unless ICSI was indicated. Indications for ICSI included severe oligozoospermia and obstructive azoospermia. Fertilization was checked 16-18 h later. Embryos were cultured to cleavage stage. Cleavage embryos with ≥6 cells and < 20% fragmentation on day 3 were defined as good quality.

On the day of oocytes retrieval, peripheral venous blood and follicular fluid were collected. The first tube follicular fluid without blood contamination was collected. The remaining follicular fluid from the same patient was pooled. After centrifugation, the supernatant follicular fluid from first tube without blood contamination was collected. And the cell pellet from the pooled follicular fluid was resuspended in PBS for further dispersing in 0.1% hyaluronidase (Sigma Chemical Co, St-Louis, Mo). Dispersed cells were then centrifuged on Ficoll gradient to remove contaminating red blood cells. Purified granulosa cells were either frozen for later RNA extraction in − 80°C or cultured in DMEM/Hams F12 containing 10% FBS (Gibco, Grand Island, NY).

### Examination of hormonal profiles

Hormonal profiles of recruited patients were determined by measuring follicular stimulating hormone (FSH, catalog number 07027346190), luteinizing hormone (LH, catalog number 07027575190), testosterone (T, catalog number 07027915190) and testerone (T, catalog number 07027915190) with chemiluminescence (Beckman Access Health Company, Chaska, MN). Anti-mullerian hormone (AMH) in the serum was determined by ELISA assay (Kangrun, Guangzhou, China, catalog number K-1401-100 N).

### Determination of insulin sensitivity in granulosa cells from PCOS and non-PCOS patients

Six patients from each group (PCOS and non-PCOS patients with or without IR) were randomly chosen for this study. Granulosa cells from these patients were cultured overnight, and Akt phosphorylation and glucose uptake were then determined following insulin (100 nM, sigma) treatment for 15 min and 30 min. Akt phosphorylation was measured with Western blotting. For glucose uptake measurement, granulosa cells cultured in a 12-well plate were placed in glucose-free DMEM medium for treatment with insulin (100 nM) for 30 min. Then, the cells were incubated with 74 kBq [F18]-fluorodeoxyglucose (^[F18]^-FDG) at 37^°C^ for 10 min. After washing with PBS, the cells were lysed with 5% NaOH. The lysate was countered along with a standard solution with a γ-counter (LS6500, Beckerman Coulter). The incorporation of ^[18F]^-FDG was quantified by the percentage of the original concentration and normalized to the number of cells as an indicator of glucose uptake.

### Measurement of SAA1 abundance in PCOS and non-PCOS patients with and without IR

SAA1 abundance in serum, follicular fluid and granulosa cells obtained from PCOS and non-PCOS patients with and without IR was measured with an immunoassay kit (R&D System, Minneapolis, MN, DY3019-05) following a protocol provided by the manufacturer. The detection limit was 1.6 ng/ml, and the cross reactivity to SAA2 was 2.7%. SAA1 mRNA abundance in granulosa cells was measured with quantitative real time PCR (qRT-PCR).

### Study of SAA1 expression in granulosa cells

SAA1 expression was examined in cultured granulosa cells prepared from non-PCOS patients with immunofluorescent staining, qRT-PCR and immunoassay. For immunofluorescent staining, cultured granulosa cells were fixed with 4% paraformaldehyde and permeabilized with 0.2% trixonX-100. After blocking the non-specific binding sites, the cells were incubated with primary antibody against SAA1 (1:100) (Abcam, Cambrige, UK) overnight at 4°C. Following washing with PBS, the cells were incubated with Alexa Fluor 488-labeled (green color) secondary antibody in darkness for 2 h. Nuclei were counterstained with DAPI (blue color). The staining was examined using a fluorescence microscope (Zeiss, DB, Germany).

For examination of SAA1 expression and production in the presence and absence of pro-inflammatory factor, granulosa cells were treated with interleukin-1β (IL-1β) (0 and 1 ng/ml, 24 h) (Sigma). After treatment, granulosa cells were collected for RNA extraction for measurement of SAA1 mRNA with qRT-PCR and conditioned culture medium was collected for determination of SAA1 production with an immunoassay kit (R&D System). Effect of SAA1 (0, 0.01, 0.1 and 1 μg/ml, 24 h) (endotoxin *<* 0.1 ng/μg, PeproTech Inc, Rocky Hill, NJ) on its own expression was also examined. SAA1 has been shown to bind TLR2/4 and activate NF-κB signaling pathway [11, 12, 28]. The role of TLR2/4 and NF-κB in the effect of SAA1 expression was investigated by treating the cells with SAA1 (1 μg/ml, 24 h) in the presence or absence of TLR2/4 antagonist OxpAPC (30 μg/ml, Invivogen, San Diego, CA) and NF-κB inhibitor JSH-23 (10 μM, Sigma) respectively.

### Examination of effects of SAA1 on insulin sensitivity in granulosa cells

Dose-dependent effects of SAA1 (0, 0.01, 0.1 and 1 μg/ml, 24 h) on PTEN and GLUT4 abundance were examined in cultured granulosa cells. Then, effects of SAA1 on insulin (100 nM, 15 min or 30 min)-stimulated Akt phosphorylation, GLUT4 translocation (from cytoplasm to membrane) and glucose uptake were examined in granulosa cells pretreated with SAA1(1 μg/ml, 24 h). The involvement of TLR2/4 and NF-κB was studied by treating the cells with SAA1 (1 μg/ml, 24 h) in the presence or absence of OxpAPC (30 μg/ml) or JSH-23 (10 μM). Time-course of SAA1 (1 μg/ml, 0, 15, 30, 60 and 180 min) on p65 phosphorylation, a subunit of NF-κB, was also examined.

### Extraction of RNA and analysis with qRT-PCR

Total RNA was extracted from frozen and cultured granulosa cells using a total RNA kit (OMEGA Bio-Tek, Norcross, GA). After determination of RNA quality, mRNA was reverse-transcribed to cDNA using PrimeScript® RT Master Mix (TaKaRa, Dalian, China). Amount of *SAA1* mRNA was determined with qRT-PCR using the above reverse-transcribed cDNA and power SYBR Premix Ex Taq™ (TaKaRa) following a previously described protocol [29]. Abundance of mRNA in each sample was calculated using the 2^-ΔΔCT^ method, and the ratio of *SAA1* transcript over *ACTB* transcript, the house-keeping gene, was obtained to indicate relative *SAA1* mRNA abundance. Primer sequences for PCR were as follows: *SAA1*, forward 5′-TTTCTGCTCCTTGGTCCTGG-3′ and reverse 5′-CTCTGGCATCGCTGATCACT-3′; *ACTB*, forward 5′-GGGAAATCGTGCGTGACATTAAG-3’and reverse 5′-TGTGTTGGCGTACAGGTCTTTG-3′.

### Extraction of protein and analysis with Western blotting

Total cellular protein was extracted from treated granulosa cells in ice-cold radioimmunoprecipitation assay (RIPA) buffer (Active Motif, Carlsbad, CA) containing protease inhibitor cocktail (Sigma) and phosphatase inhibitor (Active Motif). For GLUT4 translocation assay, cytoplasmic and membrane protein fractions were separated using a membrane and cytoplasmic extraction kit (Sheng gong, Shanghai, China). Protein sample (30 μg) was electrophoresed in 10% SDS-polyacrylamide gel and transferred to a nitrocellulose blot. After blocking the non-specific binding sites with non-fat milk, the blot was incubated with antibodies to PTEN (1:800) (Abcam), p-Akt ^ser473^ (1:2000) (Cell Signaling, Danvers, MA), total Akt (1:2000) (Cell Signaling), GLUT4 (1:1000) (Abcam), p-p65^ser536^ (1:1000) (Cell Signaling) and total p65 (1:1000) (Cell Signaling) at 4 °C overnight. After washing, the blot was further incubated with corresponding secondary antibodies conjugated with horseradish peroxidase (Sigma) for 1 h. Enhanced chemiluminescent detection system (Millipore, Billerica, MA) was then applied for band detection. To control sampling error, the same blot was probed with an antibody against the house keeping protein GAPDH (1:1000) (Santa Cruz Biotechnology, Santa Cruz, CA). For GLUT4 translocation study, the blot was incubated with antibodies against GAPDH and Na^+^-K^+^-ATPase (1:10000) (Cell Signaling) as cytoplasm and cell membrane protein markers respectively. The ratio of target protein over GAPDH or Na^+^-K^+^-ATPase was obtained to indicate relative abundance of the target protein.

### Statistical analysis

All the data were presented as mean ± SEM. The Kolmogorov-Smirnov test was used to determine whether the continuous variables were of normal distribution. Paired or unpaired Student T-test or One-way ANOVA test followed by Student-Newman-Keuls test were used to assess the differences in normally distributed variables. All data analysis was performed with the Statistical Package for Social Science (SPSS, version 18.0 for windows). *P* < 0.05 was considered as statistically significant.

## Results

### Clinical characteristics and cycle outcomes of recruited PCOS and non-PCOS patients

Table 1 illustrates clinical characteristics and cycle outcomes of recruited patients. There were no significant differences in the age, basal FSH and fasting glucose among these four groups, but higher serum levels of AMH, LH, LH/FSH, T and more retrieved oocytes were observed in PCOS patients than in non-PCOS patients regardless of IR status. White blood cells (WBC) counts were in normal range and similar in four groups, while CRP level was relative higher in PCOS patients with IR than non-PCOS patients without IR. BMI, blood fasting insulin level and HOMA-IR were higher in IR groups than in non-IR groups regardless of PCOS status. The percentage of IVF or ICSI was comparable in four groups. While the rates of fertilization, cleavage and high-quality embryo were significantly lower in PCOS with IR group than non-PCOS without IR. However, these rates were comparable among PCOS without IR and non-PCOS with or without IR.

**Table 1:** Clinical characteristics and cycle outcomes of recruited subjects with PCOS or without PCOS

	Non-PCOS without IR	Non-PCOS with IR	PCOS without IR	PCOS with IR
No.	22	22	22	22
Age (y)	29.6 ± 0.93	29.8 ± 0.81	29.8 ± 0.7	28.1 ± 0.96
BMI (kg/m^2^)	20.7 ± 0.41	26 ± 0.45 ^*#^	23.7 ± 0.66	26.7 ± 0.85^*#^
WBC (counts*10^9^)	5.6 ± 0.4	6.8 ± 0.3	6.3 ± 0.4	7.2 ± 0.4^*^
CRP (mg/L)	0.3 ± 0.4	1.1 ± 0.2	1.6 ± 0.5	2.2 ± 0.5^*^
Basal FSH (mIU/ml)	6.1 ± 0.6	6.1 ± 0.45	5.8 ± 0.3	5.5 ± 0.5
Basal LH (mIU/ml)	4.5 ± 0.47	3.9 ± 0.45	8.5 ± 0.94 ^*&^	7.1 ± 0.98 ^*&^
LH/FSH	0.7 ± 0.07	0.7 ± 0.1	1.5 ± 0.14 ^*&^	1.2 ± 0.14 ^*&^
T (nM/L)	0.7 ± 0.1	0.85 ± 0.12	1.1 ± 0.16 ^*&^	1.2 ± 0.34^*&^
AMH (ng/ml)	3.8 ± 0.4	4.6 ± 0.3	11.1 ± 1.4 ^*&^	8.7 ± 1.1^*&^
Fasting glucose (mM/L)	5.1 ± 0.07	5.4 ± 0.13	4.8 ± 0.14	5.2 ± 0.12
Fasting insulin (IU/ml)	4.8 ± 0.69	14 ± 1.05 ^*#^	6.8 ± 0.53	18.9 ± 2.8 ^*#^
HOMA-IR	1.1 ± 0.15	3.3 ± 0.24 ^*#^	1.43 ± 0.11	4.3 ± 0.61^*#^
No. of oocytes retrieved	12.6 ± 1.9	12.1 ± 1.6	18.6 ± 2 ^*&^	20.1 ± 3.3 ^*&^
IVF percentage (%)	50	62.5	62.5	56.25
ICSI percentage (%)	50	37.5	37.5	43.75
Fertilization rate	0.78 ± 0.04	0.72 ± 0.04	0.75 ± 0.04	0.63 ± 0.05 ^*^
Cleavage rate	0.99 ± 0.008	0.99 ± 0.009	0.98 ± 0.01	0.96 ± 0.02 ^*^
High-quality embryo rate	0.65 ± 0.08	0.59 ± 0.07	0.57 ± 0.06	0.41 ± 0.05 ^*^

Data are mean ± SEM values

* *P* < 0.05 vs. Non-PCOS without IR. ^&^ *P* < 0.05 vs. Non-PCOS with IR. ^#^ *P* < 0.05 vs. PCOS without IR

### Impaired insulin signaling and sensitivity in granulosa cells from PCOS patients with IR

**Fig. 1:**
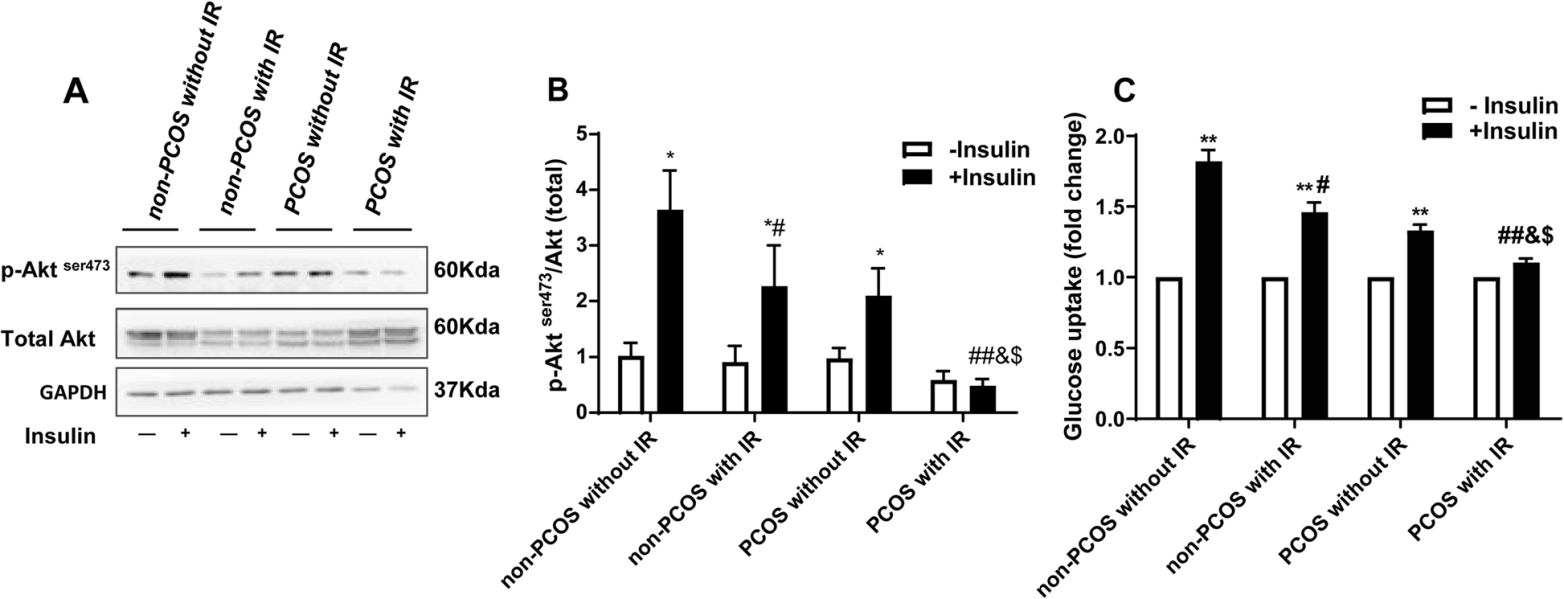
Impairment of insulin signaling pathway in granulosa cells from PCOS patients with IR. **A, B** Akt^ser473^ phosphorylation after insulin stimulation (100 nM for 15 min) in granulosa cells from non-PCOS and PCOS patients with or without IR (*n* = 6 per group). **C** Glucose uptake after insulin stimulation (100 nM for 30 min) in granulosa cells from non-PCOS and PCOS patients with or without IR (*n* = 6 per group). All values are the mean ± SEM. IR, insulin resistance. * *P* < 0.05, ** *P* < 0.01 compared with without insulin stimulation in each group. # *P* < 0.05, ## *P* < 0.01 compared with non-PCOS without IR with insulin stimulation. & *P* < 0.05 compared with non-PCOS with IR with insulin stimulation. $ *P* < 0.05 compared with PCOS without IR with insulin stimulation

Insulin (100 nM, 15 min for Akt phosphorylation and 30 min for glucose uptake) increased Akt phosphorylation and glucose uptake significantly in granulosa cells from all other three groups but not from PCOS with IR group (Fig. 1A-C), although non-PCOS with IR group also presented significantly less Akt phosphorylation and glucose uptake than non-PCOS without IR group upon insulin stimulation (Fig. 1A - C). These data indicate that impaired insulin signaling and sensitivity are present in granulosa cells from both IR groups regardless of PCOS status, but appear to be more severe in PCOS with IR group.

### SAA1 abundance in serum, follicular fluid and granulosa cells in PCOS patients

**Fig. 2:**
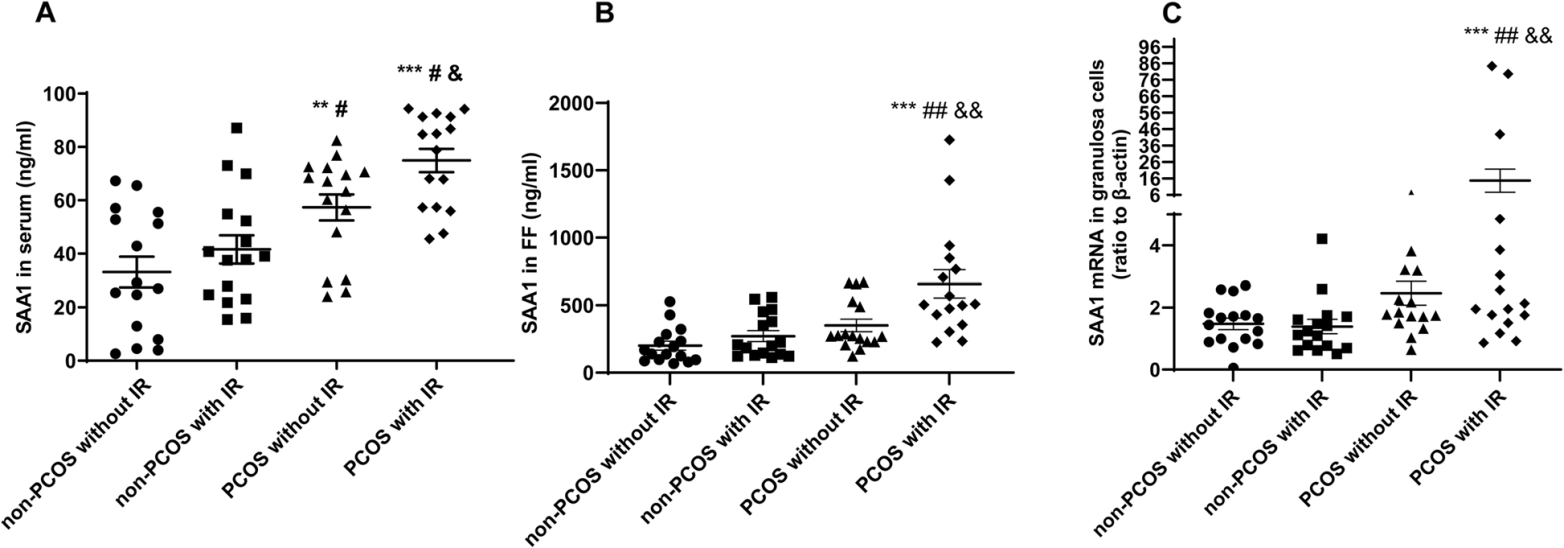
Changes of SAA1 abundance in the serum (**A**), follicular fluid (**B**) and granulosa cells (**C**) in non-PCOS and PCOS patients with or without IR (*n* = 16 per group). All values are the mean ± SEM. FF, follicular fluid. ** *P* < 0.01, *** *P* < 0.001 compared with non-PCOS without IR. # *P* < 0.05, ## *P* < 0.01 compared with non-PCOS with IR. &*P* < 0.05, & & *P* < 0.01 compared with PCOS without IR

Serum concentration of SAA1 was significantly higher in PCOS patients than in non-PCOS patients regardless of IR status with the highest concentration observed in PCOS with IR group (Fig. 2A). Similarly, SAA1 concentration in follicular fluid was also highest in PCOS with IR group but there was no difference among other three groups (Fig. 2B). Noteworthy, SAA1 concentration in the follicular fluid was 10-fold higher than that in the serum, suggesting that granulosa cells may be an alternative source of SAA1 in the follicular fluid (Fig. 2A, B). Consistently, there was increased SAA1 mRNA abundance in granulosa cells from PCOS patients with IR (Fig. 2C).

### Expression and production regulation of SAA1 in granulosa cells

**Fig. 3:**
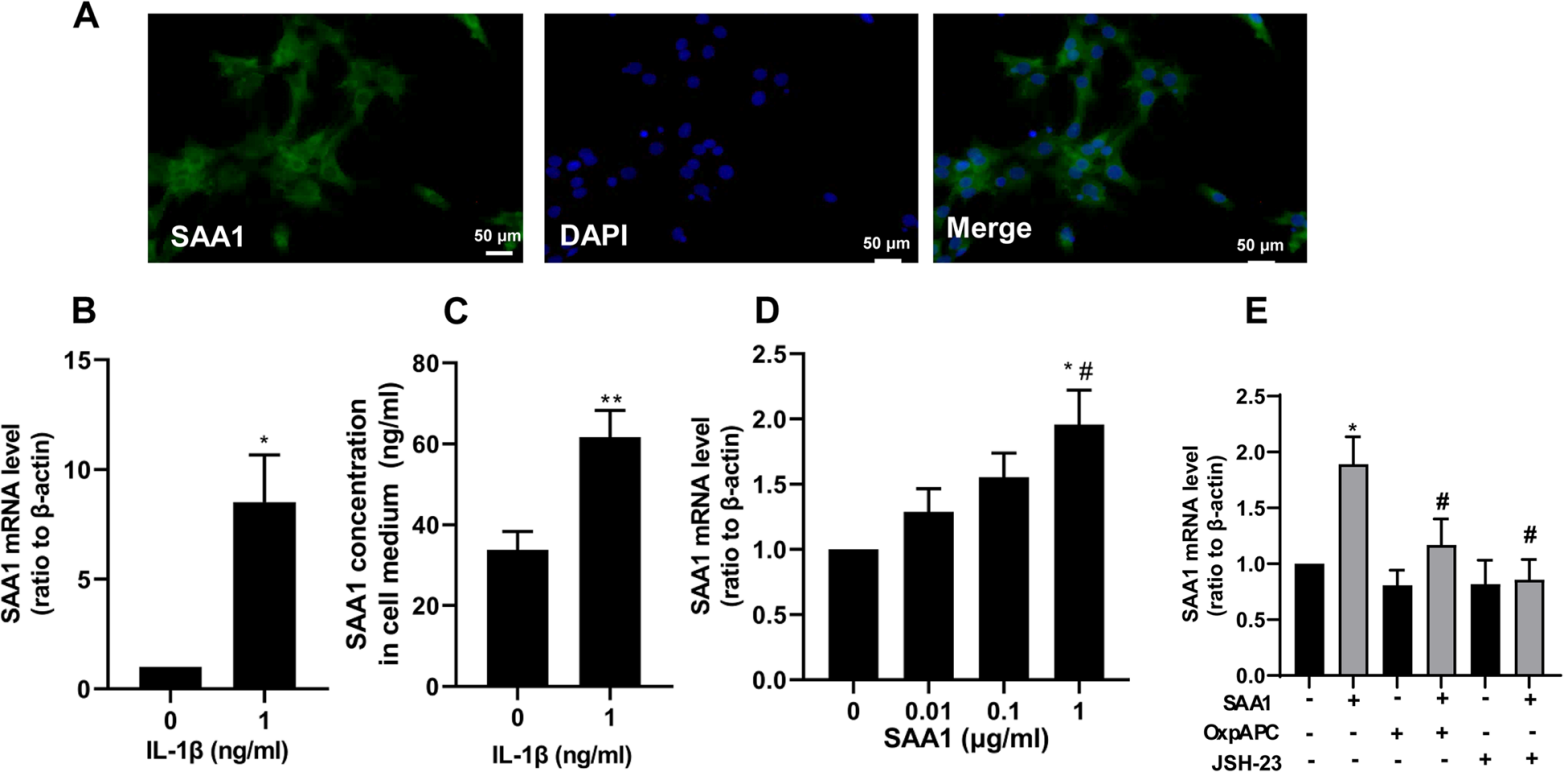
Expression and production regulation of SAA1 in human granulosa cells. **A** Immunofluorescence staining of SAA1 (green) in cultured granulosa cells. Nucleus was stained with DAPI (blue). Scale bars, 50 μm. **B, C** Effects of IL-1β (1 ng/ml for 24 h) on SAA1 mRNA expression (**B**) and secretion (**C**) (*n* = 4). * *P* < 0.05, ** *P* < 0.01 compared with control (0). **D** Concentration dependent effect of SAA1 (0, 0.01, 0.1 and 1 μg/ml for 24 h) on SAA1 expression in cultured granulosa cells (*n* = 4). * *P* < 0.05 compared with control (0), # *P* < 0.05 compared with SAA1 (0.01 μg/ml). **E** Effects of OxpAPC (30 μg/ml), a TLR2/4 inhibitor and JSH-23 (10 μM), a p65 inhibitor on the induction of SAA1 expression by SAA1 (1 μg/ml) per se (*n* = 3). * *P* < 0.05 compared with control (0), # *P* < 0.05 compared with SAA1. All values are the mean ± SEM

We further examined expression and production regulation of SAA1 in cultured granulosa cells. Immunofluorescent staining showed that SAA1 protein was present in the cytoplasm of granulosa cells (Fig. 3A). Immunoassay demonstrated basal SAA1 production in cultured granulosa cells with the concentration of 33.8 + 4.6 ng/ml over 24 h incubation (Fig. 3C). Proinflammatory cytokine IL-1β (1 ng/ml, 24 h) increased SAA1 mRNA abundance and secretion significantly in granulosa cells (Fig. 3B, C). SAA1 (1 μg/ml, 24 h) per se also increased SAA1 mRNA abundance significantly (Fig. 3D), which was blocked by either TLR2/4 inhibitor OxpAPC (30 μg/ml) or NF-κB inhibitor JSH-23 (10 μM) (Fig. 3E). These results indicate that a feedforward de novo SAA1 synthesis is present in granulosa cells, which is inducible by pro-inflammatory factors.

### Effects of SAA1 on insulin signaling pathway and glucose uptake in granulosa cells

**Fig. 4:**
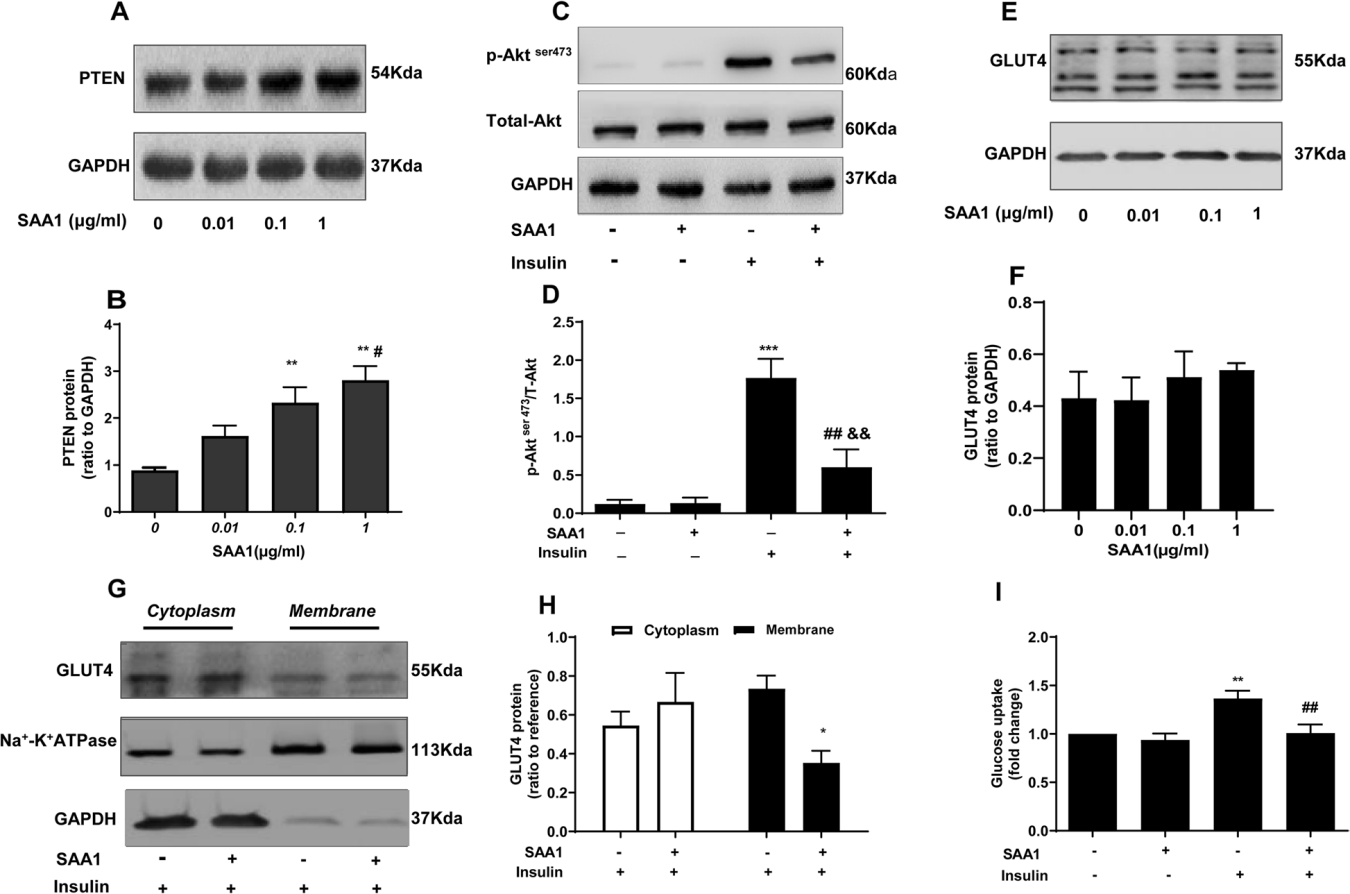
Effects of SAA1 on insulin signaling pathway in cultured granulosa cells. **A, B** Concentration dependent effect of SAA1 (0, 0.01, 0.1 and 1 μg/ml for 24 h) on PTEN expression (*n* = 4). ** *P* < 0.01 compared with ctr (0) and # *P* < 0.05 compared with SAA1 (0.01 μg/ml). **C, D** Effect of prior treatment with SAA1 (1 μg/ml for 24 h) on Akt phosphorylation induced by insulin (100 nM for 15 min) (*n* = 4). *** *P* < 0.001 compared with control-insulin, ## *P* < 0.01 vs. SAA1-insulin, && *P* < 0.01 vs. control+insulin. **E, F** Dose-dependent effect of SAA1 (0, 0.01, 0.1 and 1 μg/ml for 24 h) on total GLUT4 expression (*n* = 4). **G, H** Effect of prior treatment with SAA1 (1 μg/ml for 24 h) on GLUT4 translocation (from cytoplasm to membrane) induced by insulin (100 nM for 30 min) (*n* = 3). * *P* < 0.05 compared with control. **I** Effect of prior treatment with SAA1 (1 μg/ml for 24 h) on glucose uptake induced by insulin (100 nM for 30 min) (*n* = 4). ** *P* < 0.01 compared with control-insulin, ## *P* < 0.01 compared with control+insulin. All values are the mean ± SEM

SAA1 (0, 0.01, 0.1 and 1 μg/ml, 24 h) treatment increased PTEN abundance in a dose-dependent manner in granulosa cells (Fig. 4A, B). Pretreatment with SAA1 (1 μg/ml, 24 h) attenuated insulin (100 nM, 15 min)-induced Akt phosphorylation (Fig. 4C, D). Although SAA1 pretreatment had no effect on total GLUT4 protein (Fig. 4E, F), it attenuated insulin (100 nM, 30 min)-induced GLUT4 translocation from cytoplasm to membrane in granulosa cells (Fig. 4G, H). Consistently, glucose uptake stimulated by insulin (100 nM, 30 min) was also significantly decreased by SAA1 pretreatment (Fig. 4I). These data suggest that increased SAA1 abundance may contribute to impaired insulin signaling in granulosa cells in PCOS.

### Roles of TLR2/4 and NF-κB in SAA1-induced IR in human granulosa cells

**Fig. 5:**
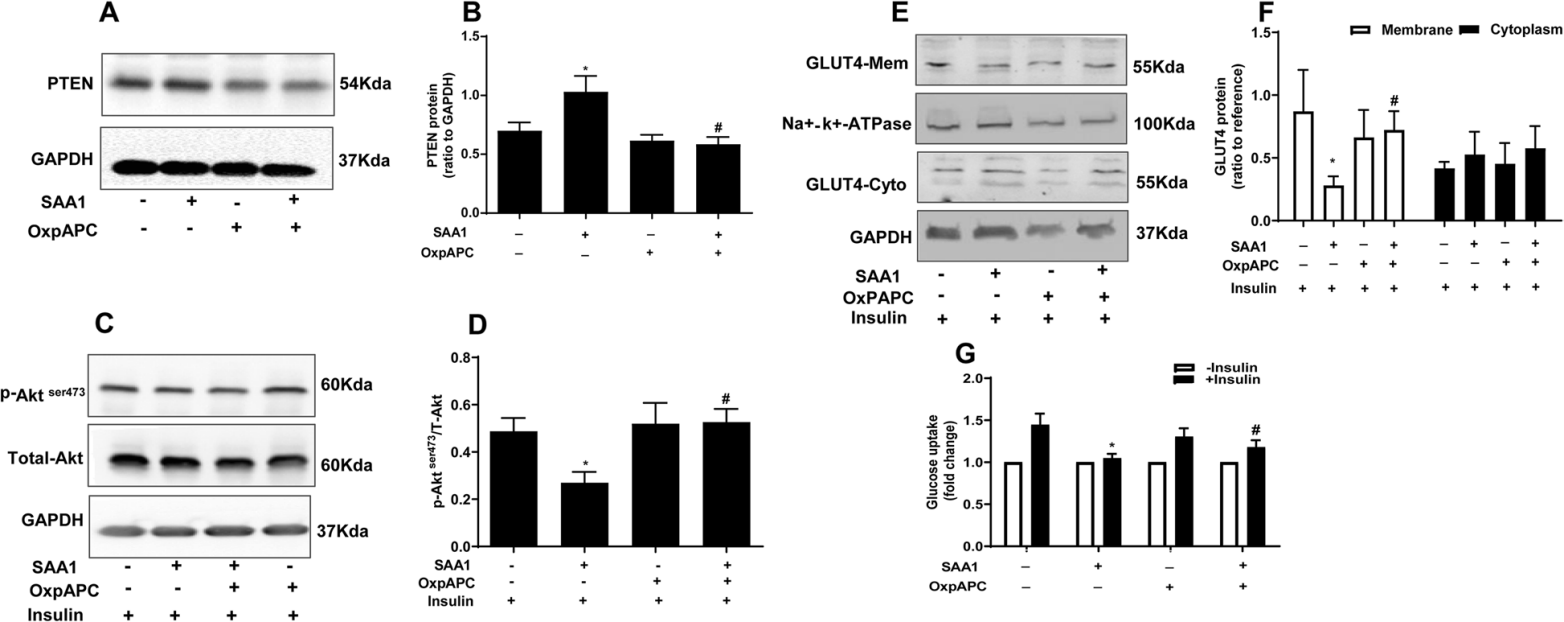
Role of TLR2/4 in SAA1-induced insulin resistance in cultured granulosa cells. **A, B** Effect of OxpAPC (30 μg/ml), a TLR2/4 inhibitor, on SAA1 (1 μg/ml for 24 h)-induced PTEN protein expression (*n* = 3). **C, D** Effect of OxpAPC on SAA1(1 μg/ml for 24 h)-attenuated Akt phosphorylation induced by insulin (100 nM for 15 min)(*n* = 4). **E, F** Effect of OxpAPC (30 μg/ml) on SAA1 (1 μg /ml for 24 h) -attenuated GLUT4 translocation induced by insulin (100 nM for 30 min) (*n* = 4). **G** Effect of OxpAPC (30 μg/ml) on SAA1 (1 μg /ml for 24 h) -attenuated glucose uptake induced by insulin (100 nM for 30 min) (*n* = 3). All values are the mean ± SEM. * *P* < 0.05 compared with control, # *P* < 0.05 compared with SAA1

**Fig. 6:**
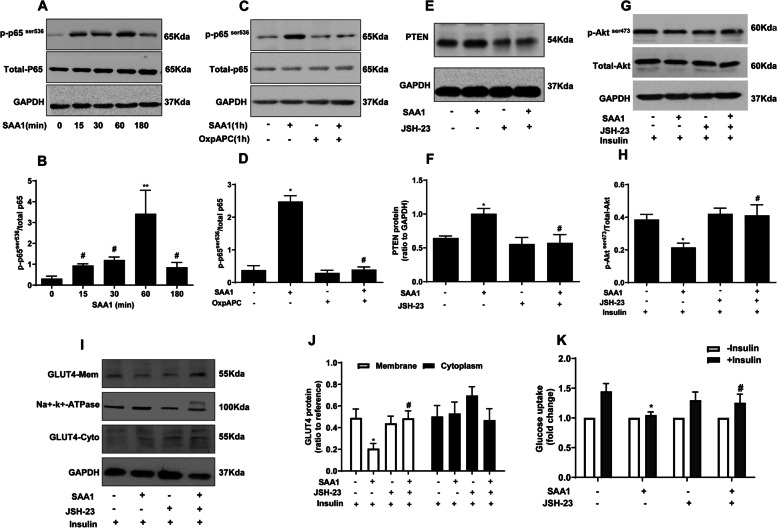
Involvement of p65 in SAA1-induced insulin resistance in cultured granulosa cells. **A, B** Effect of SAA1 (1 μg/ml for 15, 30, 60 and 180 min) on the phosphorylation of p65 (*n* = 4). ** *P* < 0.01 compared with ctr (0), # *P* < 0.05 compared with 60 min. **C, D** Effect of OxpAPC (30 μg/ml) on SAA1 (1 μg/ml for 60 min) induced p65 phosphorylation (*n* = 4). * *P* < 0.05 compared with control, # *P* < 0.05 compared with SAA1. **E, F** Effect of JSH-23 (10 μM), a p65 inhibitor, on SAA1 (1 μg/ml for 24 h) induced PTEN expression (*n* = 3). * *P* < 0.05 compared with control, # *P* < 0.05 compared with SAA1. **G, H** Effect of JSH-23 (10 μM) on SAA1 (1 μg/ml for 24 h) -attenuated Akt phosphorylation induced by insulin (100 nM for 15 min) (*n* = 4). * *P* < 0.05 compared with control, # *P* < 0.05 compared with SAA1. **I, J** Effect of JSH-23 (10 μM) on SAA1 (1 μg /ml for 24 h) -attenuated GLUT4 translocation induced by insulin (100 nM for 30 min) (*n* = 3). ** *P* < 0.05 compared with control, # *P* < 0.05 compared with SAA1. **K** Effect of JSH-23 (10 μM) on SAA1 (1 μg /ml for 24 h) -attenuated glucose uptake induced by insulin (100 nM for 30 min) (*n* = 4). * *P* < 0.05 compared with control, # *P* < 0.05 compared with SAA1. All values are the mean ± SEM

TLR2/4 inhibitor OxpAPC (30 μg/ml) blocked the induction of PTEN abundance, attenuation of insulin-induced Akt phosphorylation, GLUT4 translocation and glucose uptake by SAA1 (1 μg/ml) (Fig. 5A-G). SAA1 activated p65 phosphorylation, a subunit of the NF-κB complex in a time-dependent manner (15 min, 30 min, 60 min and 180 min) in cultured granulosa cells with the maximal effect observed at 60 min (Fig. 6A, B). When the cells were treated with SAA1 (1 μg/ml) and OxpAPC (30 μg/ml) together, the SAA1-induced p65 phosphorylation was completely abolished by OxpAPC (Fig. 6C, D). Moreover, the NF-κB inhibitor JSH-23 (10 μM) could block the induction of PTEN abundance and inhibition of insulin-stimulated Akt phosphorylation, GLUT4 translocation as well as glucose uptake by SAA1 (1 μg/ml) (Fig. 6E-K). These data indicate that SAA1 is involved in IR of granulosa cells via binding to TLR2/4 and subsequent activation of NF-κB pathway in granulosa cells of PCOS with IR.

## Discussion

The present study has demonstrated that human ovarian granulosa cells are capable of de novo SAA1 synthesis, which is inducible by proinflammatory factors including IL-1β and SAA1 itself. SAA1 overproduction may contribute to IR development in granulosa cells in PCOS patients via stimulation of TLR2/4 and NF-κB pathway.

Although SAA1 mRNA and protein have been revealed to be present in the ovary with in situ hybridization and immunohistochemical staining in a previous study [18], little is known about the expression and production regulation of SAA1 in the ovary. Our study demonstrated for the first time that human granulosa cells are not only capable of SAA1 production but are also inducible by proinflammatory factors. Of interest, SAA1 per se can induce SAA1 expression in granulosa cells as well, which is consistent with the finding in amnion fibroblast cells [30]. As such, a feedforward expression of SAA1 may develop under chronic inflammatory conditions. Chronic low-grade inflammation is believed to be present in PCOS ovary with increased abundance of a number of proinflammatory factors including IL-1β [31–33]. Since TLR2/4 and NF-κB are involved in the induction of SAA1 expression by SAA1, it is likely that SAA1 synthesis can be induced by other proinflammatory factors which utilize similar signaling pathway. In this study, IL-1β was also found to induce SAA1 production in granulosa cells. This feedforward pattern SAA1 expression under chronic inflammatory conditions may explain why there is SAA1 accumulation in granulosa cells and follicular fluid in PCOS patients with IR since patients with IR may experience more severe chronic inflammation.

In this study, we found that SAA1 concentration in the follicular fluid was about 10-fold higher than its concentration in the peripheral blood, suggesting that there may be alternative sources of SAA1 for the follicular fluid in addition to the blood. Given the abundant SAA1 expression in granulosa cells, we believe that granulosa cells are an important source of SAA1 for the follicular fluid. Since SAA1 is a small molecule which can cross the blood-follicle barrier [34], serum SAA1 may also be a source for SAA1 in the follicular fluid. Since our present study demonstrated that there was increased SAA1 abundance in both granulosa cells and blood in PCOS with IR [35, 36], we believe that the increases in SAA1 in the follicular fluid in PCOS with IR may derive from both granulosa cells and blood.

Till now, the biological functions of SAA1 in the acute phase are still not fully understood. SAA1 has been shown to be involved in the inflammatory process by promoting immune cells migration and cytokine/chemokine production [37, 38]. SAA1 has also been linked to the development of IR in adipose tissue in obesity and type 2 diabetes [14, 15]. Though metformin was routinely prescribed for patients with IR to improve metabolic disorders in our reproductive center, insulin sensitivity of granulosa cells from PCOS and non-PCOS patients with IR, including insulin-induced Akt phosphorylation and glucose uptake were still abnormal as compared with patients without IR. Of note, insulin resistance was more severe in PCOS patients with IR than non-PCOS patients with IR. Previous studies have also demonstrated that metformin treatment may not completely restore insulin sensitivity of granulosa cells [7, 39]. These indicated that some other effectors such as SAA1 involved in the development of IR in granulosa cells in PCOS patients. Our study has demonstrated that SAA1 is a contributor to IR development in the ovarian granulosa cells in PCOS as well by disrupting insulin signaling via stimulating PTEN expression and inhibiting Akt phosphorylation and GLUT4 translocation. Given the important roles of granulose cells in steroidogenesis and oocyte development [40, 41], IR in granulosa cells may distort these functions in PCOS. The observation of lower rates of fertilization and cleavage and low-quality embryo in PCOS patients with IR in this study may thus be linked, at least in part, to SAA1-induced IR in granulosa cells. A previous study has also demonstrated that elevated follicular SAA level is associated with decreased pregnancy rate [18].

Of interest, serum and follicular SAA1 concentration were higher in IR groups than non-IR groups. While BMI was also higher in IR groups than non-IR groups. As the matter of fact, we found that SAA1 concentration in the serum and follicular fluid was slightly elevated in both non-PCOS and PCOS with BMI > 24 kg/m^2^, which may be associated with obesity (Supplemental Table 1). However, we found serum and follicular fluid SAA1 concentration was higher in PCOS patients than that in non-PCOS patients when BMI was matched (Supplemental Table 2). This indicated that PCOS was a risk factor for the elevated SAA1 in serum and follicular fluid. Moreover, we found that serum SAA1 concentration increased in PCOS patients without IR as well, although not as much as PCOS patients with IR. These findings suggest that SAA1 may be involved in pathogenesis of other PCOS dysfunctions in addition to IR. SAA1 have been identified to play a role in extracellular matrix remodeling in the fetal membranes thereby participating in membrane rupture [28]. Since extracellular matrix plays important roles in the normal development of the follicle and ovulation [33, 42, 43], it would be of interest to examine whether SAA1 is also involved in extracellular matrix remodeling in the ovary and its abnormal function is linked with anovulation in PCOS in the future.

## Conclusions

In conclusion, we report a novel local feedforward production of SAA1 in ovarian granulosa cells in this study. Excessive SAA1 production under chronic low-grade inflammation reduces insulin sensitivity and contributes to IR development in granulosa cells through induction of PTEN expression and consequent inhibition of Akt phosphorylation, GLUT4 translocation and glucose uptake in PCOS patients. These effects of SAA1 were mediated by TLR2/4 with subsequent activation of NF-κB signaling. These observations support the hypothesis that SAA1 plays a crucial role in the pathogenesis of PCOS by altering insulin sensitivity and that elevated SAA1 may be an early biomarker and therapeutic target for PCOS.

## Supplementary Information

**Additional file 1: Table S1.** Serum and FF SAA1 concentration in non-PCOS and PCOS patients with overweight. **Table S2.** Serum and FF SAA1 concentration in PCOS and non-PCOS patients when BMI matched

## Data Availability

The original data presented in the study are included in the article. Further inquiries can be directed to the corresponding authors.

## Abbreviations

AMH: Anti-mullerian hormone
E_2_: Estradiol
FSH: Follicular stimulating hormone
GLUT4: Glucose transporter 4
HOMA-IR: Homeostasis model assessment of insulin resistance
IVF: In vitro fertilization
IL-1β: Interleukin-1β
ICSI: Intracytoplasmic sperm injection
IR: Insulin resistance
NF-κB: Nuclear factor kappa light chain enhancer of activated B
PCOS: Polycystic ovary syndrome
PTEN: Phosphatase and tensin homolog deleted on chromosome 10
qRT-PCR: Quantitative real time PCR
PCOS: Polycystic ovary syndrome
SAA1: Serum amyloid A1
TLR2/4: Toll-like receptor 2 and 4
T: Testosterone
