# Supplementary material for: Elevated SAA1 promotes the development of insulin resistance in ovarian granulosa cells in polycystic ovary syndrome
Source: Reprod Biol Endocrinol. 2022 Jan 3;20:4. doi: 10.1186/s12958-021-00873-3 (PMC8721971; doi:10.1186/s12958-021-00873-3)
Supplement: Supplementary file 1 — Additional file 1: Table S1. Serum and FF SAA1 concentration in non-PCOS and PCOS patients with overweight. Table S2. Serum and FF SAA1 concentration in PCOS and non-PCOS patients when BMI matched [file 12958_2021_873_MOESM1_ESM.docx]

**Table S1**. Serum and FF SAA1 concentration in non-PCOS and PCOS patients with overweight

|  | Non-PCOS | | P value | PCOS | | P  value |
| --- | --- | --- | --- | --- | --- | --- |
| BMI | ≤24 | ＞24 |  | ≤24 | ＞24 |  |
| N | 16 | 16 |  | 12 | 20 |  |
| BMI (kg/m^2^) | 20.7±0.4 | 26.1±0.5 | <0.001 | 21.8±0.4 | 27±0.5 | 0.032 |
| Serum SAA1(ng/ml） | 36.2±7.2 | 44.3±7.1 | 0.432 | 59.2±7.3 | 72.3±4.3 | 0.107 |
| FF SAA1 | 234.4±52.8 | 363.4±84.4 | 0.212 | 409.2±79.3 | 523.6±99.9 | 0.445 |

FF: follicular fluid

**Table S2.** Serum and FF SAA1 concentration in PCOS and non-PCOS patients when BMI matched

|  | BMI≤24 | |  | BMI＞24 | |  |
| --- | --- | --- | --- | --- | --- | --- |
|  | non-PCOS | PCOS | P value | non-PCOS | PCOS | P value |
| N | 16 | 12 |  | 16 | 20 |  |
| BMI (kg/m^2^) | 20.7±0.4 | 21.8±0.4 | 0.072 | 26.1±0.45 | 27±0.54 | 0.23 |
| Serum SAA1(ng/ml） | 36.2±7.2 | 59.1±7.3 | 0.039 | 44.3±7.2 | 72.2±4.3 | 0.001 |
| FF SAA1 (ng/ml) | 234.4±52.8 | 409.2±79.3 | 0.048 | 363.4±84.4 | 523.6±99.9 | 0.001 |

FF: follicular fluid
